# P-789. Urinary Tract Infections in Outpatient Men: An Analysis of Clinicians’ Diagnostic and Treatment Patterns

**DOI:** 10.1093/ofid/ofaf695.1000

**Published:** 2026-01-11

**Authors:** Tyler Brehm, Larissa Grigoryan, Laura Dillon, Trenton M Haltom, Barbara W Trautner

**Affiliations:** Baylor College of Medicine, Houston, TX; Baylor College of Medicine, Houston, TX; Michael E. DeBakey VA Medical Center / Baylor College of Medicine, Houston, Texas; Baylor College of Medicine, Houston, TX; Baylor College of Medicine, Houston, TX

## Abstract

**Background:**

There is limited evidence to guide the diagnosis and treatment of urinary tract infections (UTIs) in men. In this study, we characterized clinicians’ diagnostic and treatment decisions for outpatient men with UTIs.

**Table 1:**
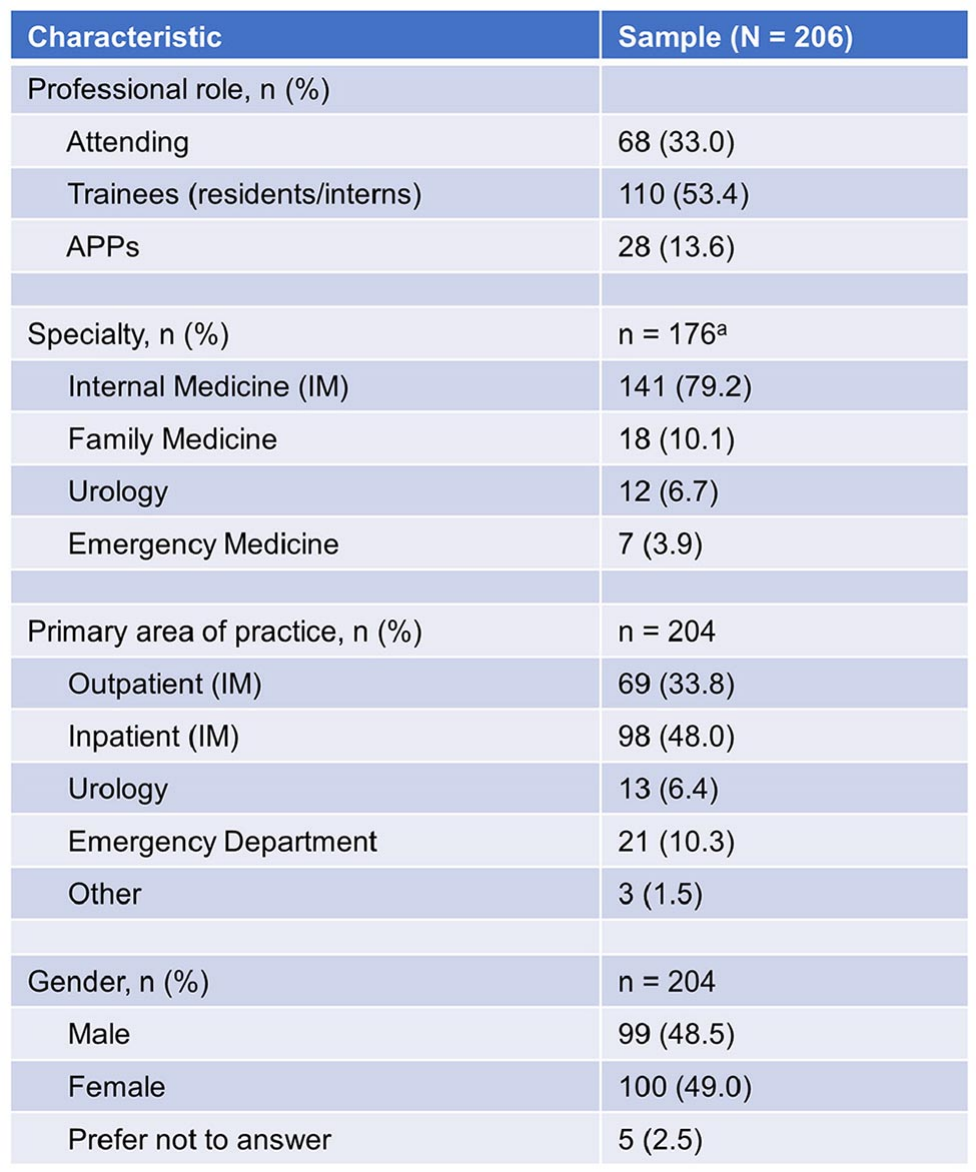
Clinician Characteristics Characteristics of clinicians who responded to the survey. APP = advanced practice professional; IQR = interquartile range; IM = internal medicine. a APPs excluded.

**Figure:**
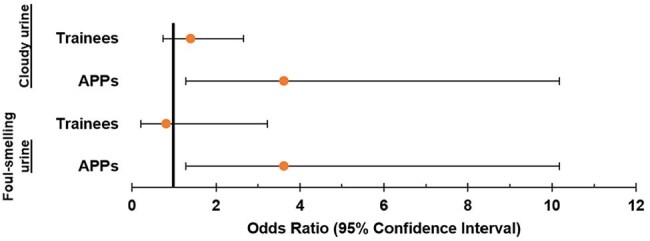
Characteristics Predicting Use of Misleading UTI Signs – Professional Role Multivariate analysis with attendings used as the reference range. APPs were significantly more likely to view cloudy (Odds Ratio [OR] 3.623, Confidence Interval [CI] 1.289-10.179, p = 0.015) and foul-smelling urine (OR 2.933, CI 1.047-8.220, p = 0.041) as indicative of UTI. APP = advanced practice professional. N = 196 (cloudy urine), N = 199 (foul-smelling).

**Methods:**

We surveyed clinicians on their diagnostic and treatment approaches to men with UTIs. Surveys were distributed to primary care and emergency medicine providers, urologists, and internal medicine residents. We analyzed clinician characteristics associated with identifying cloudy or foul-smelling urine as suggestive of UTI via logistic regression.

**Figure:**
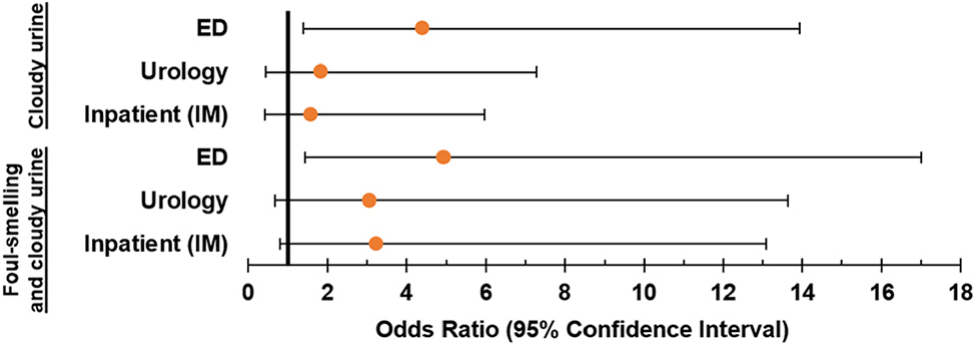
Characteristics Predicting Use of Misleading UTI Signs – Primary Area of Practice Multivariate analysis with outpatient (IM) used as the reference range. Clinicians practicing primarily in the ED were more likely to find cloudy (OR 4.935, CI 1.433-16.998, p = 0.011) and both cloudy and foul-smelling urine indicative of UTI (OR 4.411, CI 1.398-13.924, p = 0.011). ED = emergency department; IM = internal medicine. N = 196 (cloudy urine), N = 196 (both).

**Figure:**
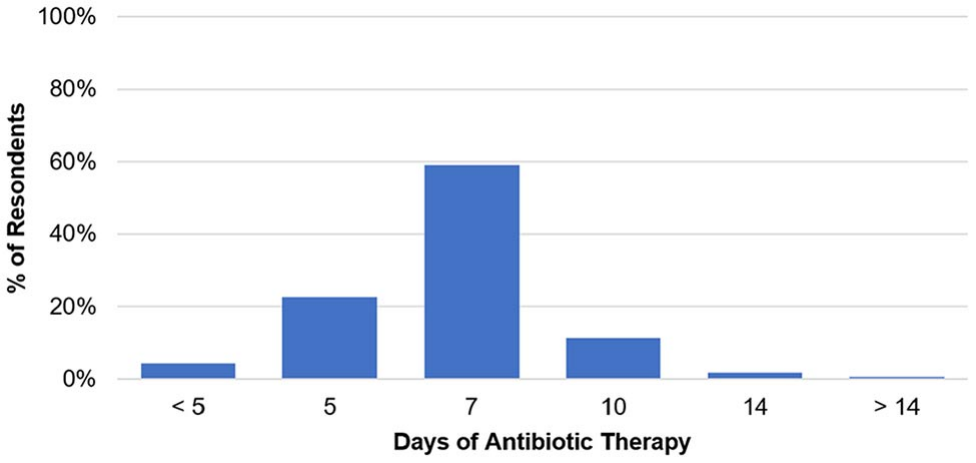
Preferred Antibiotic Therapy Duration (N = 193) The preferred duration of therapy for outpatient men with UTIs according to survey respondents.

**Results:**

Respondents (N=206) were trainees (53%), attendings (34%), and advanced practice professionals (APPs; 14%). Most physicians were internal medicine trained (79%), with a smaller proportion of family medicine (10%), urology (7%), or emergency medicine (4%). The most commonly reported symptoms and signs indicative of UTI were dysuria (96%) and suprapubic tenderness (91%). Most respondents also reported cloudy (59%) and foul-smelling urine (63%) as indicative of UTI. Across areas of practice, emergency department clinicians were significantly more likely than outpatient internal medicine clinicians to view cloudy or foul-smelling urine as indicative of UTI (Odds Ratio [OR] 4.07, Confidence Interval [CI] 1.32-12.52, *p*=0.01). Across professional roles, APPs were significantly more likely than attendings to identify cloudy urine (OR 3.62, CI 1.29-10.18, *p*=0.02) or foul-smelling urine (OR 2.93, CI 1.05-8.22, *p*=0.04) as indicative of UTI. For treatment, 87% of clinicians say they prescribe antibiotics for ≤7 days, and their most common empiric antibiotic choice was trimethoprim-sulfamethoxazole (68%).

**Conclusion:**

Clinicians found little consensus on most signs and symptoms of UTI in men. Additionally, APPs and emergency department providers significantly relied on non-evidence based, misleading signs of cloudy and foul-smelling urine. There was also a lack of treatment consensus—no one antibiotic garnered more than 68% endorsement by clinicians as an appropriate empiric choice. The variable diagnostic and treatment approaches for UTIs in men highlights the need for clinical trials in this population to guide clinical practice.

**Disclosures:**

All Authors: No reported disclosures

